# On the sensitivity of quantitative susceptibility mapping for measuring trabecular bone density

**DOI:** 10.1002/mrm.27531

**Published:** 2018-09-28

**Authors:** Maximilian N. Diefenbach, Jakob Meineke, Stefan Ruschke, Thomas Baum, Alexandra Gersing, Dimitrios C. Karampinos

**Affiliations:** ^1^ Department of Diagnostic and Interventional Radiology Technical University of Munich Munich Germany; ^2^ Philips Research Laboratory Hamburg Germany; ^3^ Department of Diagnostic and Interventional Neuroradiology Technical University of Munich Munich Germany

**Keywords:** susceptibility mapping, trabecular bone density

## Abstract

**Purpose:**

To develop a methodological framework to simultaneously measure $R_{2}^{*}$ and magnetic susceptibility in trabecularized yellow bone marrow and to investigate the sensitivity of Quantitative Susceptibility Mapping (QSM) for measuring trabecular bone density using a non‐UTE multi‐gradient echo sequence.

**Methods:**

The ankle of 16 healthy volunteers and two patients was scanned using a time‐interleaved multi‐gradient‐echo (TIMGRE) sequence. After field mapping based on water–fat separation methods and background field removal based on the Laplacian boundary value method, three different QSM dipole inversion schemes were implemented. Mean susceptibility values in regions of different trabecular bone density in the calcaneus were compared to the corresponding values in the $R_{2}^{*}$ maps, bone volume to total volume ratios (BV/TV) estimated from high resolution imaging (in 14 subjects), and CT attenuation (in two subjects). In addition, numerical simulations were performed in a simplified trabecular bone model of randomly positioned spherical bone inclusions to verify and compare the scaling of $R_{2}^{*}$ and susceptibility with BV/TV.

**Results:**

Differences in calcaneus trabecularization were well depicted in susceptibility maps, in good agreement with high‐resolution MR and CT images. Simulations and in vivo scans showed a linear relationship of measured susceptibility with BV/TV and $R_{2}^{*}$. The ankle in vivo results showed a strong linear correlation between susceptibility and $R_{2}^{*}$ (*R* ^2^ = 0.88, *p* < 0.001) with a slope and intercept of −0.004 and 0.2 ppm, respectively.

**Conclusions:**

A method for multi‐paramteric mapping, including $R_{2}^{*}$‐mapping and QSM was developed for measuring trabecularized yellow bone marrow, showing good sensitivity of QSM for measuring trabecular bone density.

## 1 INTRODUCTION

Osteoporosis remains the main clinical driver for trabecular bone MRI. It is defined as the medical condition of low bone mineral mass and density. Fractures due to osteoporotic bone loss greatly reduce individual quality‐of‐life and have an increasing prevalence in all demographic groups. In the United States and also in Europe, up to one in three post‐menopausal women is estimated to experience bone fractures due to decreased bone densities.^1^, ^2^ Osteoporosis can be treated successfully if diagnosed at an early stage: bone mineral density (BMD) measurements based on dual energy X ray absorptiometry (DEXA) are currently the gold standard for osteoporosis screening.^3^ However, BMD of healthy and osteoporotic patients overlap and have low accuracy in predicting fracture risk. Quantitative Computed Tomography (QCT) measurements allow the simultaneous assessment of BMD and bone microstructure, improving the ability to predict biomechanical bone strength and eventually fracture risk. However, QCT is associated with increased radiation dose^4^ compared to DEXA. MRI has been previously proposed and is highly desirable for osteoporosis screening, thanks to its non‐invasiveness. However, high‐resolution MR trabecular bone imaging remains limited to distal skeletal sites and is not feasible due to its low sensitivity in major osteoporosis sites like the spine.^5^

The acquisition of multiple echoes in lower resolution gradient echo MRI enables the measurements of bone marrow effective properties as an alternative way to indirectly assess trabecular bone network health. Previously, gradient echo‐based $R_{2}^{*}$‐mapping has been proposed as an indirect measure of trabecular density.^6^, ^7^, ^8^ The susceptibility difference between the bony trabeculae—showing no MR signal in normal gradient echo MRI sequences—and the MR signal generating bone marrow in the intra‐trabecular space, causes large inhomogeneities of the induced magnetic field.^6^ Such field inhomogeneities on the scale of the trabecular network lead to the dephasing of proton spins in bone marrow and consequently result in an accelerated relaxation due to intra‐voxel dephasing on a voxel scale not resolving trabeculae directly. However, the mechanism of trabecular bone growing predominantly in the direction of the greatest force load^9^ and the formation of connected rod‐like and plate‐like structures^10^, ^11^ give trabecular networks an inherently complex topology. Both,^6^ numerical simulations as well as the theoretical analyses predict that in the static dephasing regime^6^at time scales on which diffusion effects become negligible as the dephasing field inhomogeneities are much stronger than the signal decay due to diffusive motion—the intra‐voxel dephasing can be effectively described by a mono‐exponential decay with decay rate $R_{2}^{\prime }$. In addition, theoretical analysis also indicates a strong dependence of the intra‐voxel dephasing on the orientation of trabecular bone with respect to the magnetic field, the main field strength, the voxel size and the intra‐voxel distribution of bone inclusions; all effects which were also experimentally observed in previous phantom studies.^7^, ^12^, ^13^ The dependence of $R_{2}^{*}$ on all above parameters has reduced the robustness of $R_{2}^{*}$‐mapping measuring trabecular bone density in clinical applications.

Quantitative susceptibility mapping (QSM) has been emerging as a technique to measure, a fundamental tissue property, the average magnetic susceptibility per voxel, independent of field‐strength.^14^ In the past QSM has been extensively studied in the brain, resulting in numerous neurological applications, such as for example, identification of multiple sclerosis lesions,^15^, ^16^ the discrimination of cerebral micro‐bleeds and intracranial calcifications^17^ or monitoring of iron deposition.^18^ This success motivated applications of QSM outside the brain and already encouraged QSM for breast imaging,^19^ measuring liver iron content,^20^ and imaging of cortical bone.^21^ Similar to cortical bone, trabecular bone is diamagnetic and has a lower susceptibility than water, and therefore, most soft‐tissues. As the apparent transverse relaxation rate in cortical bone is very large, $R_{2}^{*}∼2500\;s^{-1}$,^22^ ultra‐short echo time MRI needs to be performed to obtain phase information inside voxels of cortical bone for reliable QSM.^21^ Voxels containing trabecular bone show MR signal due to the surrounding bone marrow and in theory, their averaged scalar magnetic susceptibility scales linearly with the ratio of bone volume to total volume (BV/TV). Following Wiedeman's additivity law, a mixture of different components constitutes a bulk magnetic susceptibility, which is the sum of the proportionate susceptibilities of each component in the mixture.^23^ Therefore, QSM is a natural candidate to indirectly measure trabecular bone density non‐invasively.

QSM reconstructs tissue magnetic susceptibility from the phase information of MRI gradient‐echo data,^24^ which involves three main conceptual steps^14^: (i) estimation of the magnetic field inside the scanner, (ii) removal of field contributions not originating from susceptibility sources inside a defined region of interest (ROI), and (iii) solving the field‐to‐susceptibility inverse problem. All three steps face technical challenges when applying QSM in the body. First, the total magnetic field needs to be estimated. In brain QSM, where typically a tight brain mask is defined as ROI, scaling of unwrapped single echo phase images,^25^ dual echo phase subtraction^26^ or voxel‐wise nonlinear fitting of a single frequency component to the phase evolution over multiple echoes^27^ is used to obtain a field map. In body QSM, however, the presence of fat needs to be accounted for as the chemical shifts of its spectral resonances cause a complex multi‐exponential phase evolution in voxels with non‐zero fat fraction.^28^ The parameter estimation problem for the field mapping step in body QSM is therefore the same as for complex‐based water–fat separation methods.^29^ However, as the field map is the primary parameter of interest it cannot be treated as a mere nuance parameter, which is often subject to (multi‐scale) smoothing in some current water–fat separation algorithms.^30^, ^31^ For the second background field removal step there are several techniques available that can be loosely categorized into two approaches: kernel‐convolution‐based methods such as the Laplacian Boundary Value method (LBV)^32^ or Sophisticated Harmonic Artifact Reduction for Phase data (SHARP)^33^ and minimum‐norm methods, such as Projection onto Dipole Fields (PDF).^34^ However, all these techniques have not been thoroughly studied in body applications. The third field‐to‐susceptibility step poses an ill‐posed, ill‐conditioned inverse problem and can be solved by means of regularization.^35^ In its Bayesian interpretation, the employment of different regularizations corresponds to the introduction of different prior knowledge about the underlying susceptibility distribution.^36^ The most common regularizer used in QSM is total variation (TV),^37^, ^38^ in combinations with or without morphlogical edges weightings^39^ and evaluated with the ℓ_1_ or ℓ_2_ norm.^40^, ^41^ TV promotes piece‐wise constant susceptibility distributions in the QSM reconstructions, whereas the recently proposed total generalized variation (TGV) allows also for more linear susceptibility variations.^42^, ^43^ The question of which regularization scheme is best for the MRI application and clinical question at hand is still subject of ongoing research^44^ across body regions and applications. The purpose of the present study is to develop a methodology for simultaneous $R_{2}^{*}$‐mapping and QSM of trabecularized bone marrow and to assess the sensitivity of QSM for measuring trabecular bone density using both numerical simulations and in vivo measurements. Some results of this work have been preliminarily reported in.^45^, ^46^, ^47^

## 2 METHODS

The feasibility of QSM for trabecular bone density mapping and its performance compared to relaxometry was evaluated in in vivo scans of the ankle region and in numerical simulations of a simplified trabecular bone model.

### 2.1 In vivo measurements

Fourteen volunteers ((35 ± 16)years) were scanned in a 3 T scanner (Ingenia, Philips, Release 5.1.8, Best, The Netherlands) after informed written consent by each volunteer and approval by the institutional review board (Klinikum rechts der Isar, Technical University of Munich, Munich, Germany).

#### 2.1.1 MR sequence parameters

A time‐interleaved multi‐gradient‐echo sequence (TIMGRE) was used to acquire complex source images of the ankle with a total of nine echoes in three acquisitions employing flyback gradients (monopolar read‐out).^48^ Using an eight‐channel foot coil, scan parameters included TR = 13 ms, TE_min_ = 1.25 ms, ΔTE = 0.7 ms, no partial Fourier encoding, flip angle = 5^∘^, orientation = sagittal, readout direction = feet–head, field of view (FOV) = 220 × 220 × 102 mm^3^, acquisition voxel size = (1.5 mm)^3^, bandwith/pixel = 1431.4 Hz, scan time = 7 minutes 30.1 s, SENSE reduction factor = 1.

Additionally, all volunteer scans included a balanced steady‐state free precession sequence (bSSFP) with two phase cycles, TR = 8.5 ms, TE = 3.4 ms, no partial Fourier encoding, scan time = 7 minutes 29.1 s and a voxel size of 0.3 × 0.3 × 0.9 mm^3^ at a slice coverage of only the calcaneus (FOV = 220 × 220 × 60 mm^3^) that was used to obtain an apparent measure of trabecular bone density.

#### 2.1.2 Post‐processing

**Figure 1:**
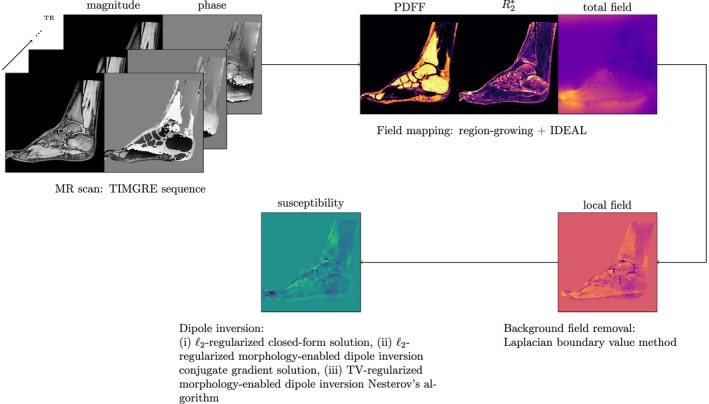
Flowchart overview of the post‐processing pipeline for quantitative susceptibility mapping for trabecular bone density mapping

The TIMGRE images were subject to the QSM postprocessing pipeline outlined in Figure 1.

First, raw k‐space data was reconstructed with MRecon.^49^ Sensitivity maps acquired in pre‐scans were used in the SENSE algorithm to combine separate coil images^50^ without any parallel imaging reduction.

To estimate the total magnetic field, a complex‐based water–fat separation algorithm assuming a known seven‐peak fat‐spectrum^28^ with a single‐$R_{2}^{*}$ ‐correction was initialized with a multi‐seed region growing scheme from.^51^ Further details connected to the voxel signal model equation are given in the Supporting Information S1. The water–fat separation employed for magnetic field mapping yielded, besides the total magnetic field, a quantitative proton‐density fat fraction map available from the complex water *W* and fat *F* results as |*F*|/|*W* + *F*| and a $R_{2}^{*}$ map.

To estimate and extract the local field from the total field map, the Laplacian boundary value method (LBV)^32^ from the MEDI toolbox^52^ was used.

To estimate a susceptibility map by performing dipole‐inversion, the following MEDI cost function^39^ regularized by Total Variation (TV) was optimized:

(1) $$\chi =\underset{\chi^{\prime }}{\text{argmin}}\left|\right|W_{d}\left(\gamma B_{0}F^{†}DF\chi^{\prime }-f_{L}\right){\left|\right|}_{2}+\lambda ||W_{g}\nabla \chi^{\prime }{\left|\right|}_{\ell_{p}},$$

where *W*_d_ was the data weighting, *F* Fourier transformation, *D* the dipole kernel in k‐space defined by $D\left(|k|\;\neq \;0\right)\;=\;1/3-k_{z}^{2}/{\left|k\right|}^{2}$ and *D*(|**k**| = 0) = 0, *f*_*L*_ the local field,^14^ and *W*_g_ the gradient weighting. Note that we assumed the main magnetic field as pointing along the *z*‐axis, $B_{0}=B_{0}\hat{z},$ γ is the proton's gyromagnetic ratio.

Three different dipole‐inversion schemes were implemented: (i) a closed form ℓ_2_‐regularized solution of (1) with *W*_d_ = *W*_g_ = 1,^40^ (ii) an optimization of the ℓ_2_‐regularized MEDI costfunc^38^ solved by the conjugate gradients method, and (iii) an optimization of the TV‐MEDI cost function^39^ solved by Nesterov's algorithm.^53^ For the two MEDI optimizations, the gradient weighting *W*_g_ was obtained by thresholding the absolute value of the forward gradient on the water–fat opposed phase image |*W*−*F*| such that 40% of the voxels in the tissue region belong to edges and are weighted by a value of 0.01, whereas all other voxels in *W*_g_ were set to 1. For the data weighting mask *W*_d_ the maximum intensity projection across echo times (MIP_TE_) was scaled to the dynamic range [0, 1]. The regularization parameter λ was chosen by visually comparing the quality of susceptibility maps from the first volunteer dataset reconstructed with a range of λ's from 0.0001 to 1 varied on a log‐scale. This selection of λ was also guided by plotting an L‐curve heuristic as the discrepancy $\left|\right|\left(F^{†}DF\chi -f_{L}\right){\left|\right|}_{2}^{2}$ after the reconstruction versus λ, which is shown in Supporting Information Figure S2.

The susceptibility maps were not subject to any referencing, the absolute range of values was unchanged for all subject datasets and the DC offset of the dipole kernel was zero.

To access the measurement of trabecular bone density in the quantitative susceptibility maps across all subjects, two ROIs inside the calcaneus were drawn in the lower‐resolution TIMGRE magnitude images of each subject where the calcaneus is known to have different BV/TV ratios, the subtalar and the tuber calcanei, both depicted in the top right of Figure 4.^7^ The third distinct region in the calcaneus with much less BV/TV, the cavuum calcanei, was not included in the ROI analysis as increased vascularization in this region complicates QSM measurements. The ROI label masks created in 3D Slicer (Version 4.7,^54^) were used to extract label statistics in the quantitative maps derived from the TIMGRE source images.

After linear registration of the bSSFP to the TIMGRE images using SimpleITK^55^ and resampling the label masks to the bSSFP orientation, a measure of apparent BV/TV was determined in each ROI by the histogram‐based double‐thresholding method described in.^56^

Measures of central tendency inside the defined ROIs were extracted for all quantitative parameters. Correlations between the mean values were investigated by linear regression.

#### 2.1.3 Computer tomography patient scans

The post‐processing of the TIMGRE scan described above was also applied for two patients (one male age 70, one female age 76) that were equally informed and asked to participate in the study as the healthy volunteers. As part of their clinical care, low‐dose whole body CT images were taken and approved to be evaluated for this work.

The calcaneus in the TIMGRE and the CT images were manually registered until complete line‐up. Besides visual comparison of CT images in Hounsfield units and the estimated parameter maps, ten ROIs were drawn in the subtalar and the tuber calcanei, respectively, for each patient dataset. Again linear regression was performed to correlate $R_{2}^{*}$ and susceptibility with CT attenuation.

### 2.2 Numerical simulations

Similar to previous work simulating magnetic fields in trabecular bone,^57^, ^58^ we forward simulated magnetic field distortions created by a simple trabecular bone model. In a cubic box of 128 × 128 × 128 voxels, spherical inclusions resembling the trabecular bone volume inside a ROI were randomly positioned in space with the possibility to overlap. The cubic ROI was centered in an empty three dimensional cube with an edge length three times as large. According to the same forward model as in Equation (1), *f*_*B*_ = γ*B*_0_ *F* ^†^ *DFχ*, the field map *f*_*B*_ was simulated with one fixed *B*_0_‐direction with varying susceptibility difference of the trabecular bone inclusions to the surrounding, Δ*χ* = (−0.5, −1.0, −1.5, −2.0) ppm, varying number of spherical inclusions, *N*=100,150,...,300, and varying radii, *r* = (5, 10, 15, 20) voxel units. All inclusions were simulated to have the same radius and susceptibility. Each combination of the parameters Δ*χ*, *N* and *r* was explored by a Monte‐Carlo program in 100 different spatial configurations of the inclusions. The program was implemented in the Python programming language (Python version 3.5.4) making use of the default random number generator from the numpy module (numpy version 1.14.2).

The spectral density function, an auto‐binned histogram of all field values outside the spherical bone inclusions, was subject to a Lorentzian fit. The full‐width at half maximum (FWHM) of the fitted Lorentzian curve was employed as a measure of the reversible relaxation rate $R_{2}^{\prime }$. To re‐invert the noise‐free field maps to susceptibility, the simple closed‐form Tikhonov‐TV regularized solution^40^ was used and the mean susceptibility value inside the cubic trabecular bone ROI was taken for comparison. The simulation resulted in two effective parameters resembling $R_{2}^{*}$ and susceptibility *χ* for the whole ROI for all configurations of spherical inclusions (of varying BV/TV) inside the box.

## 3 RESULTS

### 3.1 In vivo measurements

**Figure 2:**
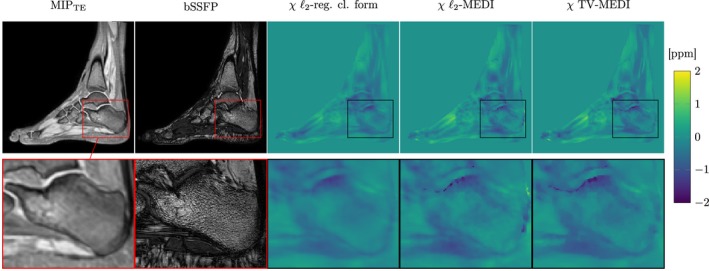
Visual comparison of different dipole inversion methods for quantitative susceptibility mapping (QSM) in the calcaneus. Column 1: maximum intensity projection over echo times in MR scan used for QSM. Column 2: high‐resolution image from a balanced steady state free‐precession (bSSFP) sequence. Column 3–5: QSM result from a ℓ_2_‐total variation regularized closed form susceptibility solution,^40^ an ℓ_2_‐total variation (TV) regularized morphology‐enabeled dipole inversion (MEDI),^38^ and a TV‐MEDI, respectively^66^

Figure 2 shows a comparison of the three different dipole inversions next to the maximum intensity projection over echo times (MIP_TE_) and the co‐registered bSSFP scan in one exemplary subject. Due to its short $R_{2}^{*}$, trabecular bone shows no MR signal in non‐UTE sequences and is only visible indirectly as the bone marrow in the intra‐trabecular space exhibits strong MR signal. As visible in the bSSFP scan in Figure 2, bottom row, second column, high trabecular density is indicated by denser black signal drop out regions like in the subtalar. In regions with less trabeculae, bone marrow fills more volume and consequently MR signal is brighter as observed in the tuber calcanei. For all dipole inversions, the susceptibility map closely follows the trabecular bone density in the calcaneus and depicts regions of varying BV/TV in the same dynamic range but with different textures. Edges in the ℓ_2_‐regularized closed form solution (third column) show up smooth and transitions between regions of higher and lower BV/TV appear continuous. In contrast the ℓ_2_‐MEDI result (fourth column) shows a lot more finer variations and subtalar and tuber calcanei areas seem to show more susceptibility variance compared to the closed form solution. The fifth column shows the TV‐regularized MEDI result in which the edges of the calcaneus are depicted more clearly compared to the closed form solution, while the variance of susceptibility in subtalar and tuber calcanei regions appears lower compared to *l*_2_‐MEDI. The same difference were observed in visual comparison of the three implemented dipole inversions in all acquired datasets; In all cases the TV‐regularized MEDI showed visually the best results in terms of homogeneous susceptibility in regions with different BV/TV, defined edges in susceptibility following the magnitude images and suppressed streaking in all orientations. TV‐MEDI also showed the highest correlations with apparent BV/TV and $R_{2}^{*}$ than the other dipole inversions in regression analysis of ROI statistics below.

The degree of smoothing and the streaking reduction is not only dependent on the chosen regularizer in Equation (1), but also on the regularization parameter λ. For each of the three different regularizers, the optimal regularization parameter was determined by comparing the visual appearance of resulting susceptibility maps while changing λ on a  log ‐scale. The computed L‐curves, showing the discrepancy $\left|\right|\left(F^{†}DF\chi_{\text{est}}\left(\lambda \right)-f_{L}\right){\left|\right|}_{2}^{2}$ versus λ, were computed for one subject and are shown in Supporting Information Figure S2. While these curves showed a local minimum in the range of λ's for all implemented dipole inversions, based on visual considerations about the greater reduction of streaking artifacts, the chosen λ's were in the vicinity of the L‐curve minimum but about one order of magnitude larger. The λ's obtained in the above way in one subjects were set in the reconstructions for all other datasets and had the following values: λ(ℓ_2_ cl. form) = 0.2, λ(ℓ_2_‐MEDI) = 0.1, λ(TV‐MEDI) = 0.03. The voxel size and FOV, which would also effect the choice of an optimal regularization parameter, were kept the same in all acquired datasets and consequently did not alter the optimality of the chosen λ's.

**Figure 3:**
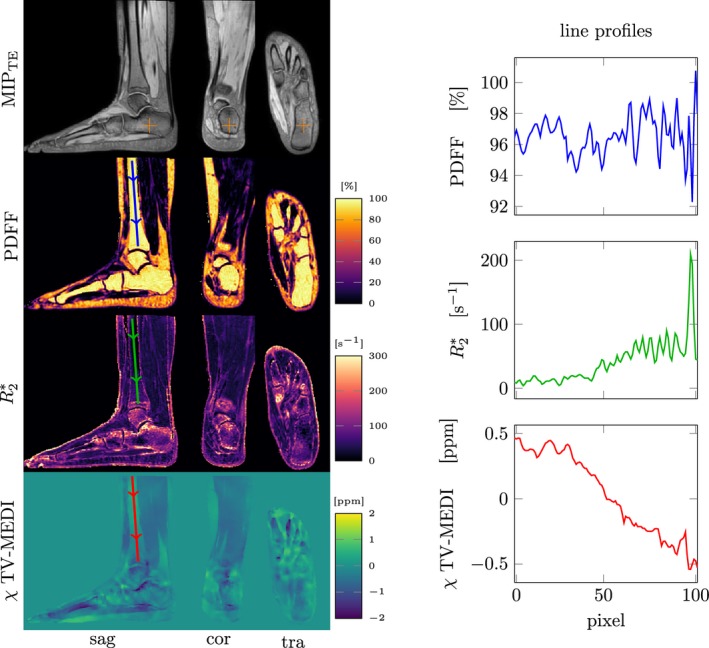
Quantitative parameter maps in an examplary ankle dataset (left) and corresponding line profiles in the distal tibia. With constant proton density fat fraction (PDFF), the transverse relaxation rate $R_{2}^{*}$ increases and the susceptibility *χ*—estimated with an ℓ_1_‐total variation (TV) regularized morphology‐enabeled dipole inversion—decreases toward the end of the tibia, where trabecularization increases

In Figure 3 one can observe the high proton‐density fat fraction close to 100% in the ankle bone marrow of another exemplary healthy volunteer dataset. Line plots in the annotated regions of linearly increasing trabecularization in the distal tibia from superior to inferior is traceable in both quantitative MR parameter maps, showing the sensitivity of QSM on trabecular bone density.

**Figure 4:**
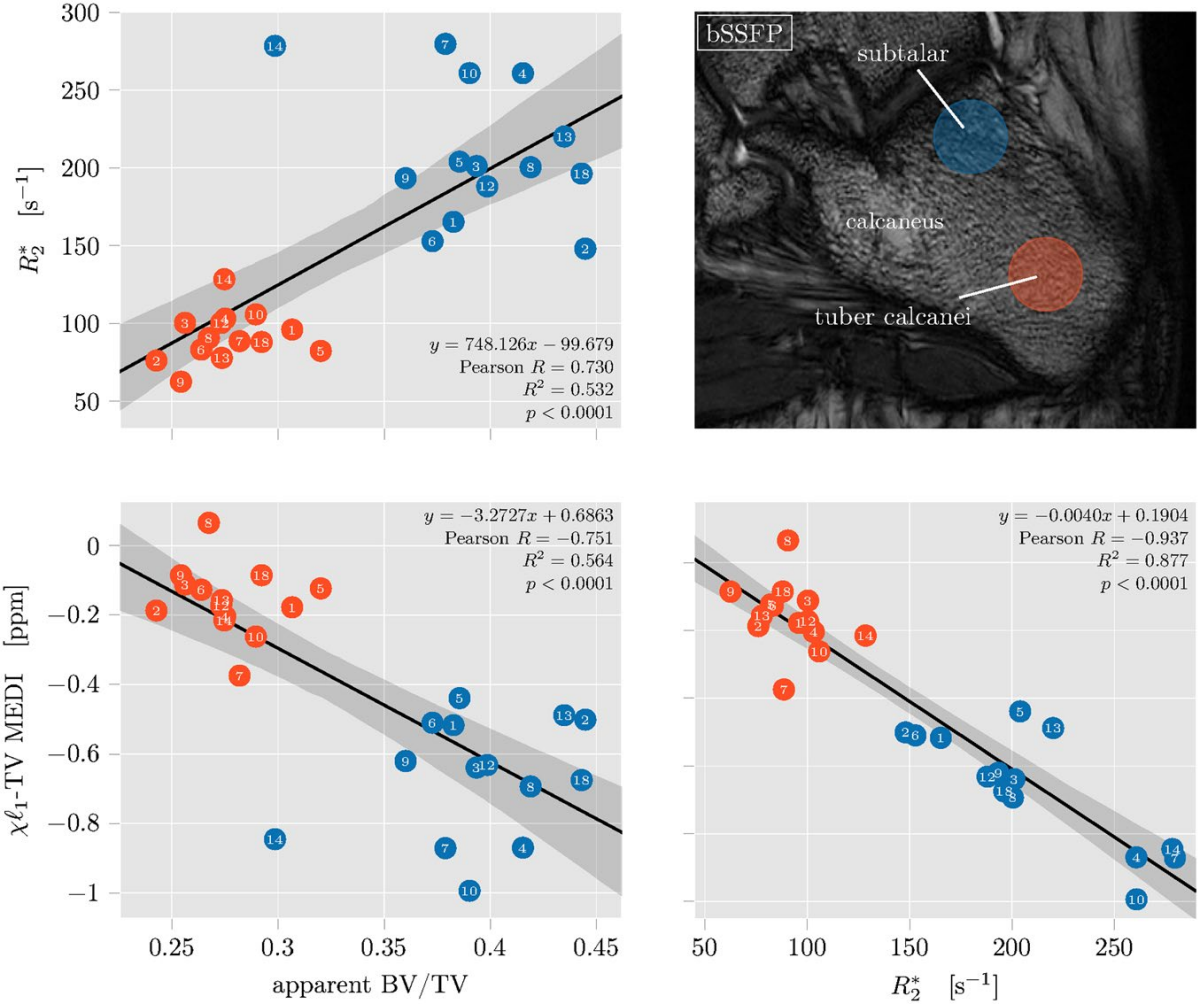
Results of regression analysis of ROI label statics for 15 ankle datasets. In each datasets two ROI's of different trabecular bone density in the calcaneus were defined—the subtalar densly trabecularized and the tuber calcanei showing less trabecularization in the high‐resolution balanced steady state free‐precession (bSSFP) scan (top right)

To be able to assess the ability of QSM to map trabecularized bone marrow regions of different BV/TV quantitatively, the mean values of apparent BV/TV from the bSSFP scan and the reconstructed $R_{2}^{*}$ and susceptibility from the TIMGRE inside two defined ROIs—subtalar and the tuber calcanei—scan were correlated. Figure 4 shows pair plots with all regression results. Mean ROI values of all parameters are clearly separated for both subtalar (blue) and tuber calcanei (red). For the two ROIs, averaged quantitative estimates for all subject datasets cluster also distinctively on all parameter axes, even though, no referencing was used before extraction of ROI statistics. The regression results confirm the expected trends of the subtalar ROI with higher BV/TV showing larger apparent BV/TV, larger $R_{2}^{*}$ and more diamagnetic (lower) susceptibility and the tuber calcanei with lower BV/TV showing lower apparent BV/TV, lower $R_{2}^{*}$ and less diamagnetic (larger) susceptibility. All correlations appear to be highly significant (*p* < 0.001) but with differences in the explainable variance. While the regression of parameters estimated with different sequences, $R_{2}^{*}$ or TV‐MEDI susceptibility versus apparent BV/TV, show mild correlations of $R^{2}\left(R_{2}^{*}\;\text{vs. app. BV/TV}\right)\;=\;0.53$ and *R* ^2^(*χ* vs. app. BV/TV) = 0.56, correlation is strong between parameters from the TIMGRE sequence as $R^{2}\left(\chi \;\;\text{vs.}R_{2}^{*}\right)\;=\;0.88$.

In the apparent BV/TV a clear outlier value from subject dataset 14 can be detected, while the overall spread of BV/TV values in all subjects is larger than for $R_{2}^{*}$ and susceptibility.

The same regression plots with susceptibility values from different dipole inversions systematically show less strong correlations between *χ* and all other parameters and are shown in the Supporting Information Figure S3.

**Figure 5:**
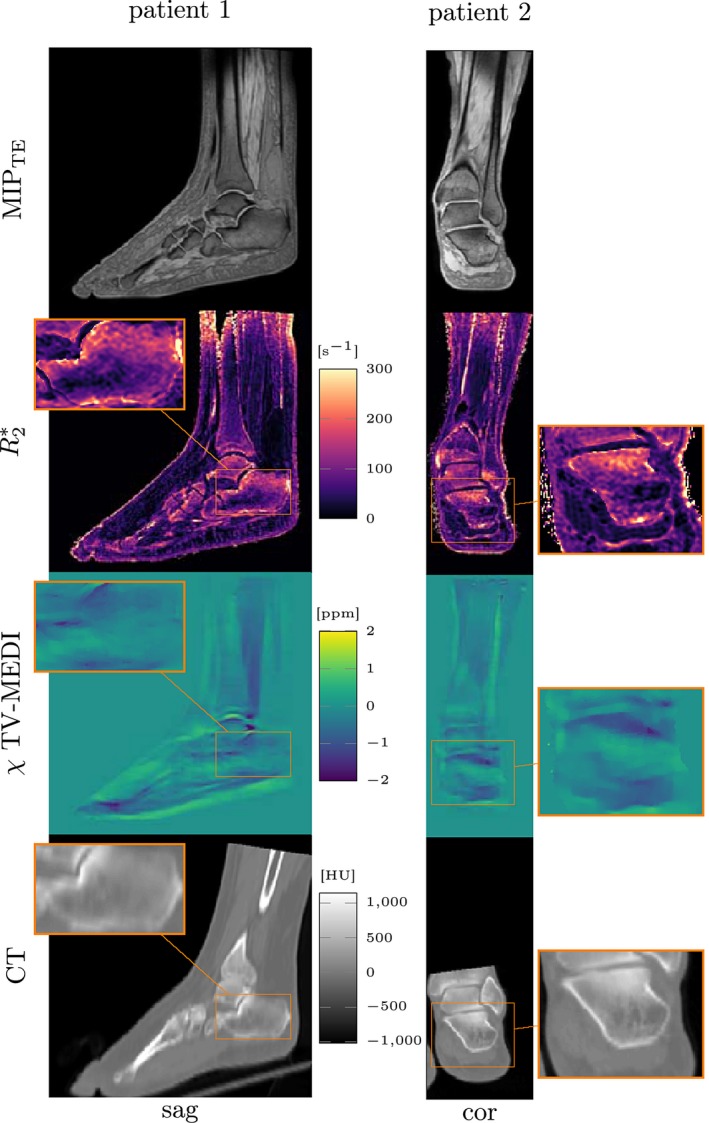
Quantitative parameter maps estimated from the MR time‐interleaved multi‐gradient echo scan compared to computed tomography (CT) available in two patient datasets. The susceptibility *χ*—estimated with an ℓ_1_‐total variation (TV) regularized morphology‐enabeled dipole inversion—depicts regions of greater trabecular density (high CT attenuation) with more diamagmetic values. An extended version of this Figure including all orientations is available as Supporting Information Figure S4

**Figure 6:**
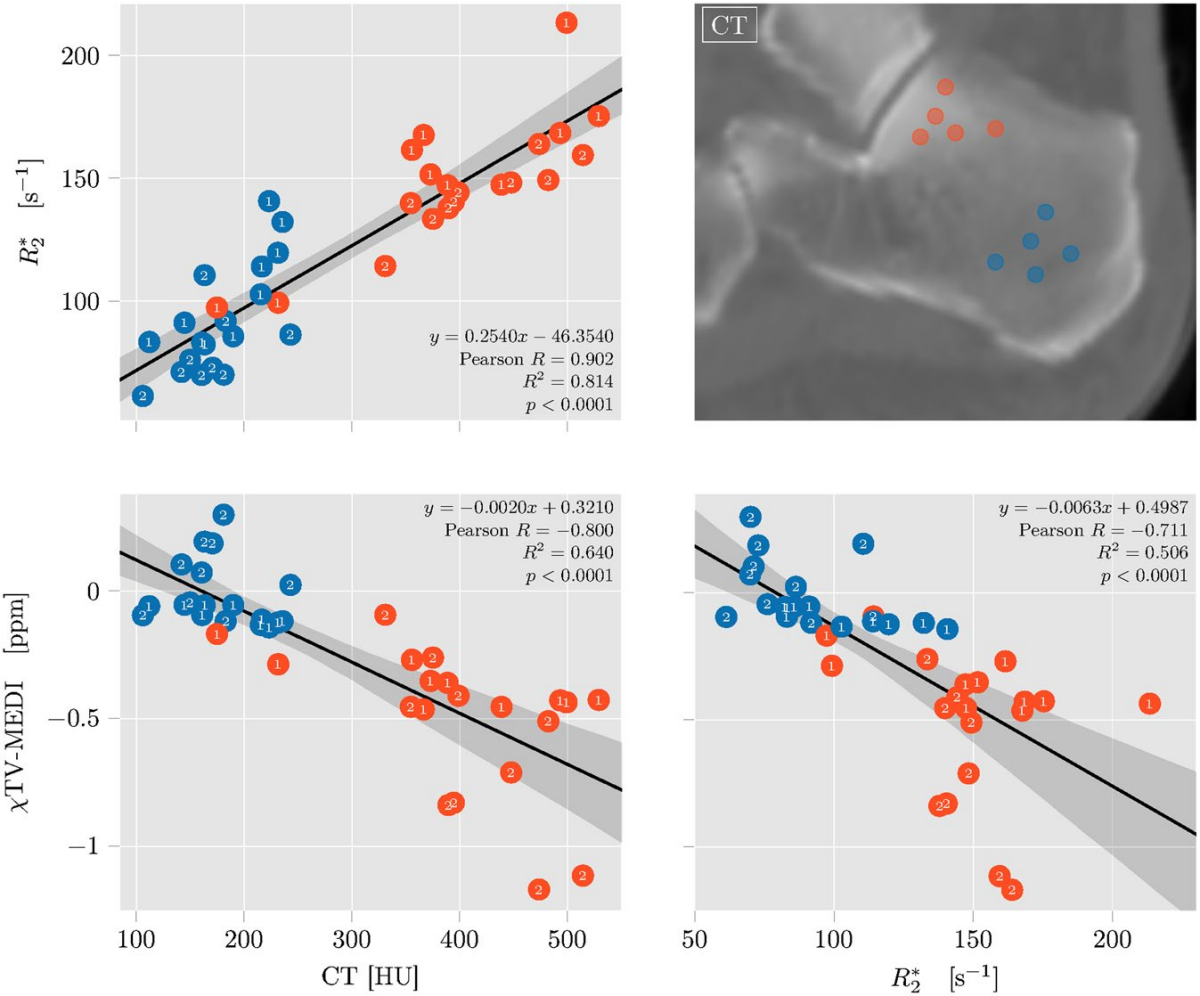
Results of regression analysis of ROI label statics in two ankle datasets in two regions of different trabecular bone density in the calcaneus—the subtalar densly trabecularized and the tuber calcanei showing less trabecularization in the high‐resolution balanced steady state free‐precession (bSSFP) scan (top right); (10 more ROI's not visible in the displayed slice)

Figure 5 clearly shows how the subtalar region with higher bone mineral mass and density exhibits the largest CT attenuation inside the calcaneus. The increased BV/TV in subtalar region also results in greater intra‐voxel dephasing indicated by larger $R_{2}^{*}$ and more diamagnetic averaged susceptibility. Due to the lower number of patient datasets compared to the number of healthy volunteer datasets, ten smaller ROIs were drawn in the patients’ subtalar and tuber calcanei regions, respectively, to be able to perform a similar regression analysis as compared to TIMGRE–bSSFP regression in the healthy volunteers above. Figure 6 shows pair plots of the TIMGRE–QCT regression results in the two patient datasets. Again, large ROI values of CT attenuation in more trabecularized regions correlate significantly with larger $R_{2}^{*}$ and lower susceptibility (*p* < 0.0001 for regressions between all parameters). While here, $R_{2}^{*}$ correlates much stronger with CT attenuation ($R^{2}\left(R_{2}^{*}\;\text{vs. CT}\right)\;=\;0.81$) than $R_{2}^{*}$ with apparent BV/TV before, the correlations of susceptibility versus CT attentuation and $R_{2}^{*}$ are weaker (*R* ^2^(*χ* vs. CT) = 0.64 and *R* ^2^(*χ* vs. CT) = 0.50).

Again, Figures showing the incorporated QSM results of the other dipole inversion are available in Supporting Information Figures S5. Similar to the TIMGRE–bSSFP comparison, TV‐MEDI performed better in visual rating and correlation to QCT than the other dipole inversions.

Close observation of the susceptibility maps in the calcaneus of patient 1 (male) and patient 2 (female) in Figure 5 shows a difference in the dynamic range. The difference of susceptibility in the subtalar and the tuber calcanei appears larger in patient 1 as in patient 2. In Figure 6 one can also observe this inter‐patient variation as the difference of *χ* values of subtalar (red) and cavuum calcanei (blue) in the regression plots of the bottom row is greater for the patient 1 than for patient 2 (see labeled points separately).

### 3.2 Numerical simulations

**Figure 7:**
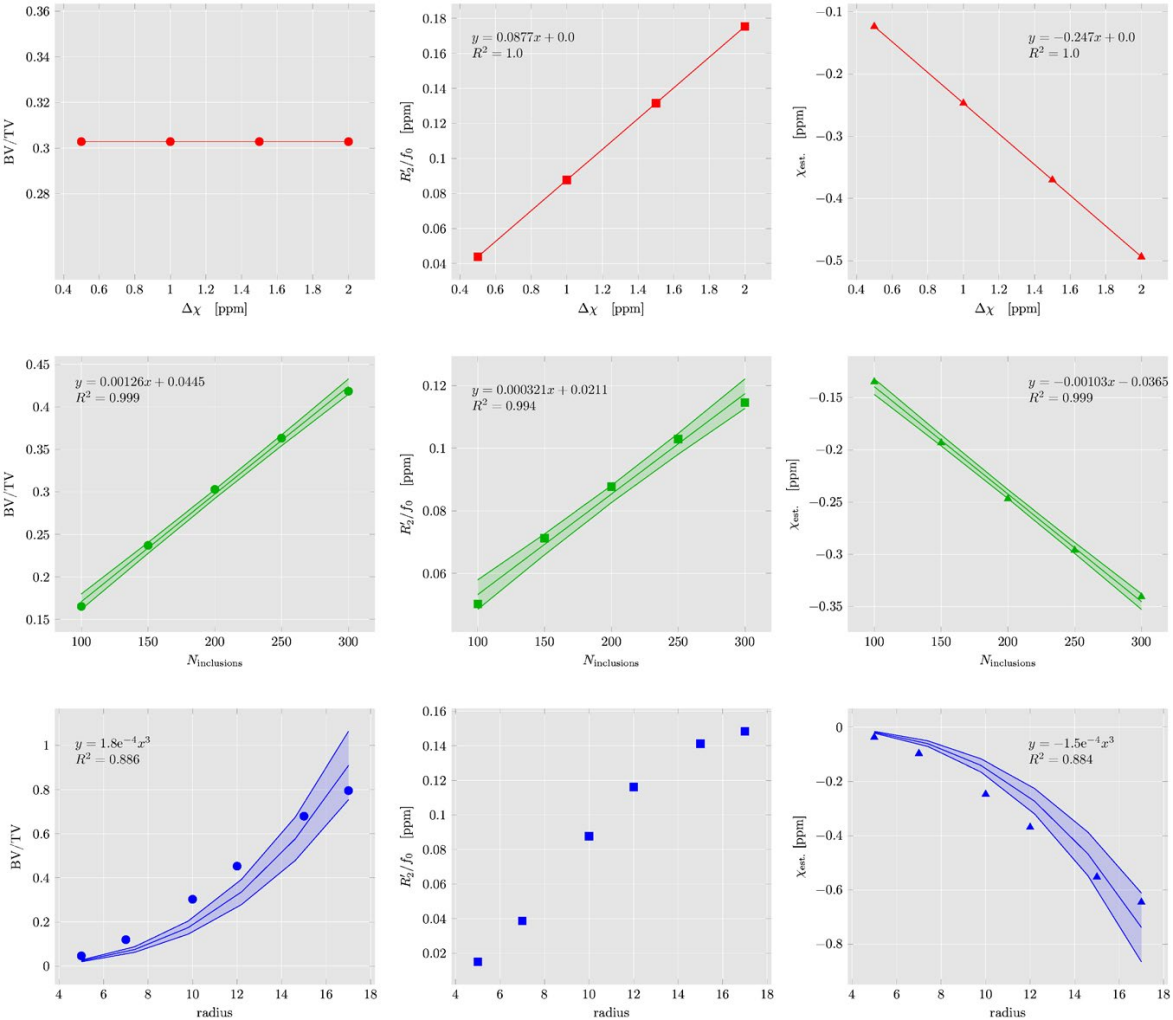
Numerical simulation results in a simplified trabecular bone model consisting of randomly located spherical bone inclusions inside a cubic ROI, with varying relative susceptibility difference to their surrounding (Δ*χ*_ext._, top row, red curves), number (*N*_inclusions_, middle row, green curves), and radius (*r*, bottom row, blue curves). Plotted are the ratio of bone volume to total volume (BV/TV, first column), the $R_{2}^{\prime }$ decay rate (FWHM of a Lorentzian fitted to the spectral density function), the dominant and reversible part of transverse relaxation rate $R_{2}^{*}$ (second column), and the mean susceptibility inside the ROI after a ℓ_2_‐total variation regularized closed form susceptibility solution

In Figure 7, the results summarizing the numerical simulations are shown. In each row two parameters of the simplified trabecular bone model of spherical inclusions are kept fixed, while the third parameter is varying—either Δ*χ* (top row, red curves), *N*_inclusions_ (middle row, green curves), or radius *r* (bottom row, blue curves). Column‐wise, from left to right, BV/TV (disc markers), $R_{2}^{\prime }$ (square markers), or mean susceptibility (triangle markers) are plotted.

When the susceptibility difference Δ*χ* is linearly increased while *N*_inclusions_ = 200 and *r* = 10, BV/TV stays constant, $R_{2}^{\prime }$ and susceptibility increase linearly as expected.

When the number of inclusions *N*_inclusions_ is increased for fixed Δ*χ* = −1 ppm and *r* = 10, both BV/TV and *χ*_est._ increase linearly with slight deviations as the spherical inclusions are allowed to overlap.

For fixed Δ*χ* = −1 ppm, *N*_inclusions_ = 200 and increasing radii of the spherical inclusions, both BV/TV and *χ*_est._ follow an expected *r* ^3^ volumetric increase, while the apparent measure on $R_{2}^{\prime }$ deviates from this trend. Again, deviations from the *r* ^3^ curve are observable due to the overlapping of bony spheres in the model.

**Figure 8:**
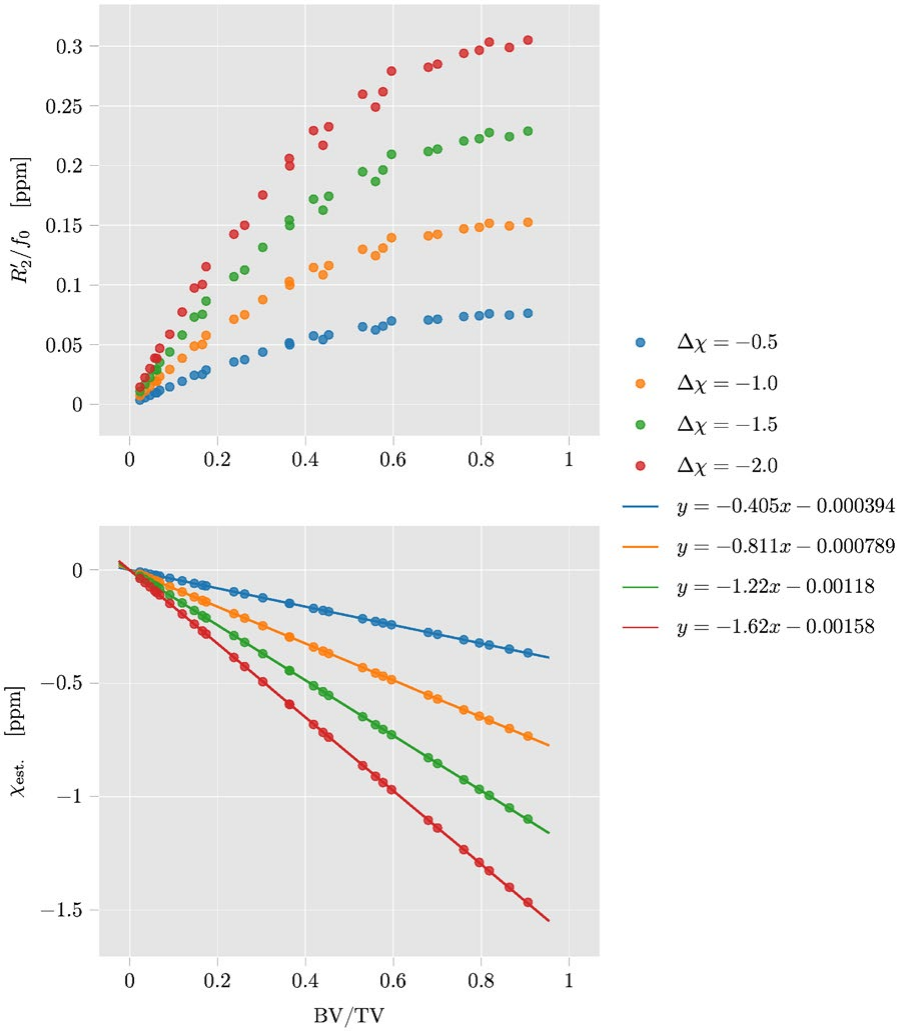
Effective parameters simulated in a simplified trabecular bone model consisting of randomly located spherical bone inclusions inside a cubic ROI plotted against the bone volume to total volume (BV/TV). Top: the $R_{2}^{\prime }$ decay rate (FWHM of a Lorentzian fitted to the spectral density function). Bottom: mean susceptibility inside the cubic ROI after a ℓ_2_‐total variation regularized closed form susceptibility solution

Figure 8 plots $R_{2}^{\prime }$ (top) and the estimated mean susceptibility *χ*_est._ (bottom) against the BV/TV over all simulated configurations of bony spheres. *χ*_est._ clearly obeys a linear relationship with the exact slope depending on the susceptibility value of the spherical inclusions with respect to the surrounding, while the $R_{2}^{\prime }$ versus BV/TV does not show a similar linear increase.

Error bars for all numerical results determined by taking the variance over all Monte‐Carlo events are negligible (smaller than the marker size in both Figures 7 and 8), which can be easily explained by the translational invariance of the spherical inclusions inside the ROI, while the occurring cropping of spheres at the ROI edges is only of minor importance as their radius is small compared to the edge length.

## 4 DISCUSSION

In this work, we addressed the feasibility of QSM for trabecular bone imaging. The presented results show a clear sensitivity of QSM on trabecular bone volume density. Visually the in vivo susceptibility maps were able to differentiate regions of different BV/TV as shown in the distal tibia (Figure 3) and the calcaneus regions, subtalar and tuber calcanei (Figure 5). Quantitative regression results of extracted ROI statistics in the calcaneus regions also confirm a good sensitivity of QSM for trabecular bone density quantification. (Figures 4 and 6). Numerical simulation of the simplified trabecular bone model of overlapping randomly distributed spherical bone inclusions inside a cubic ROI were able to show the same sensitivity of QSM on BV/TV.

In the ankle region, bone marrow almost exclusively consists of fatty yellow bone even in young healthy volunteers, visible as the almost 100% proton density fat fraction inside the bones in Figure 3. Thus, it is valid to assume there are only two components of constant susceptibility in the trabecularized bone marrow of the calcaneus, bone and fat. While bone has no MR signal at the echo times used in this study (≥ 1 ms) due to its fast relaxation, the protons in the fat molecules generate signal, which exhibits a complex phase evolution due the multiple resonances present in the fat spectrum. Effects of signal interference between fat resonances on the multi‐gradient‐echo data and therefore on the measured total field map and $R_{2}^{*}$‐map are considered in the present work as the water–fat separation algorithm takes the spectral complexity of fat into account. Hence, the contrast in the measured field map and $R_{2}^{*}$ is mainly driven by magnetic field inhomogeneities induced by magnetic surface currents at the trabeculae–bone marrow interfaces. On a voxel scale the inhomogeneities created by the susceptibility difference of the trabecular bone network to inter‐trabecular space leads to an intra‐voxel dephasing. The consequent increase of the dominant $R_{2}^{\prime }$ contribution to the apparent relaxation $R_{2}^{*}$ inside the voxel is primarily observable in the magnitude decay of the bone marrow signal.

The phase changes inside the bone marrow translate to the measured total field map. As the trabecular size is only in the range of 100–150 μm and inter‐trabecular spacing is in the range of 300–600 μm,^5^ the created field inhomogeneities are averaged over typical MR voxel sizes around 1 mm^3^ or bigger, leaving no or only a little local frequency offset due to the presence of trabeculae inside a voxel. In cylindrical trabecular bone models this total absence of a local frequency offset is theoretically derived in^6^ and in the presently studied trabecular bone model of spherical inclusions is only very small. However, as the susceptibility of bony trabeculae and bone marrow fat proportionally add up inside a voxel (due to Wiedemann's additivity law), a bulk susceptibility effect of the bone–fat mixture in the voxel is exerted on the surrounding, which scales linearly with the BV/TV inside the voxel. The averaging out of local frequency shifts from the induced field inhomogeneities created by the trabeculae‐bone marrow interfaces takes place in an arbitrarily big ROI's containing trabecular structures and the ROI's bulk susceptibility effect on the outside depends not only on the mean susceptibility but also on the shape of the ROI.^6^ In gradient‐echo MRI data the susceptibility effect inside the ROI is therefore encoded in the phase evolution of the ROI's outside. The global field‐to‐susceptibility inversion step of QSM, conceptually a deconvolution with a kernel (the dipole kernel) of equal dimension as the field map, is therefore able to estimate susceptibility differences from trabecular bone regions of different BV/TV, because the information about the bulk susceptibility effect on the rest of the FOV is present and exploitable even though theoretically the effect is absent inside of the trabecular bone.

**Figure 9:**
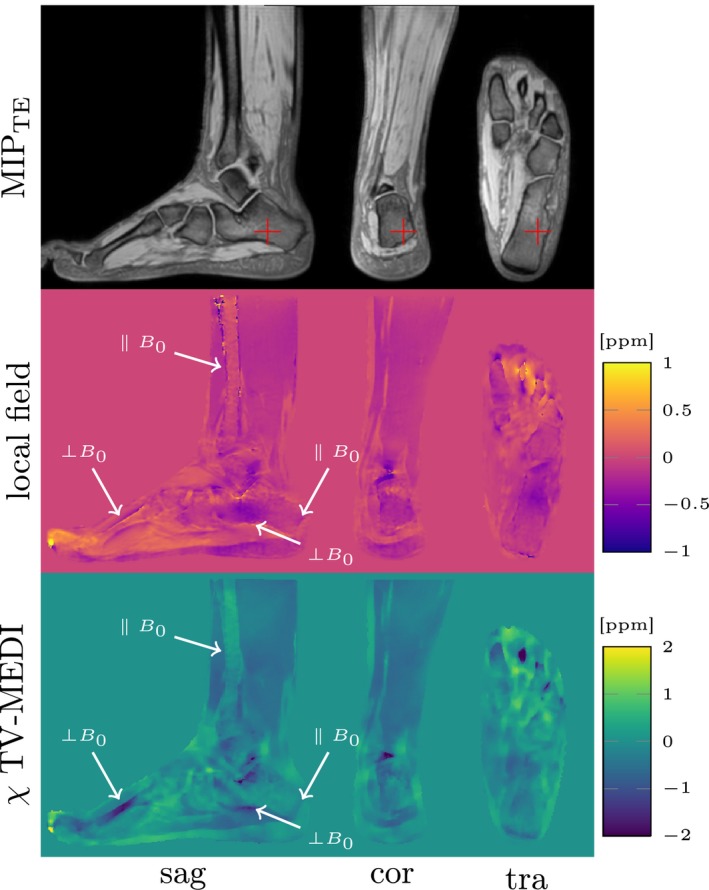
Illustration of the inablility of the proposed quantitative susceptibility mapping (QSM) pipeline to measure cortical bone structures whose edges are aligned with the main magnetic field *B*_0_, here pointing in feet–head direction. The cortical bone of the os metatarsi II in the foot is correctly assigned a diamagmetic susceptibility, because its perpendicular orientation to *B*_0_ leads to magnetic field distortions in the surrounding. In contrast the cortical bone of the distal tibia in the leg does not show the same susceptibility as its parallel orientation with respect to *B*_0_ does not lead to field distortion on its outside, which renders it invisible to QSM. In the employed MR sequence without aquiring ultra short echo times (non‐UTE) no phase information is available inside of the cortical bone regions

Sensitivity of QSM on trabecular bone density in vivo was demonstrated in the gradient echo sequence with standard echo times, where bone does not exhibit MR signal. The present work could showcase the possibility of QSM on visualizing bulk susceptibility effects of trabecularized yellow bone marrow not only in the calcaneus, but also in the distal tibia. Along the dia‐, meta‐, and epiphysis, where trabecularization increases the susceptibility profiles show increasingly diamagnetic values. While differences in trabecular bone density could be detected, cortical bone could not be imaged reliably by the complete QSM post‐processing. In the imaging volume there is no susceptibility information about thicker cortical bone structures, if their geometries do not lead to field distortions in the outside regions of the cortical bone. The fundamental limitation of QSM to detect MR invisible susceptibility inclusions that do not create local field contributions outside the inclusion can be seen in Figure 9. In the dataset shown in Figure 9, exemplary for all ankle datasets, the os meta tarsi, one of the longer bones in the foot leading to the toes clearly shows a strong diamagnetic value (dark) in the susceptibility map, while the cortical bone of the distal tibia which typically points along the main magnetic field *B*_0_ is not visible in the susceptibility map due to the lack of MR signal within the cortical bone tissue and the absence of field distortions on its outside. Consequently, for the focus on cortical bone, MR phase information in the inside of the bone is necessary to reliably estimate its susceptibility, which manifests the need for ultra‐short echo time (UTE) imaging to provide MR signal inside bone as in.^21^ In the context of traditional osteoporosis screening however, the characterization of bone loss in the trabecular bone network is of primary interest. QSM for trabecular bone imaging showed sufficient sensitivity on BV/TV based on the non‐UTE acquisition employed in this work.

We found that the exact appearance of trabecularized regions in the reconstructed susceptibility maps depends on the regularization techniques being used in the dipole inversion step of the QSM pipeline. Many different regularization techniques were proposed.^44^ In this work, we implemented and compared three different regularization schemes: a closed form solution with an ℓ_2_ total variation (TV) regularization, an ℓ_2_‐MEDI and a TV‐MEDI. By visual comparison of the TV‐MEDI approach appeared to have the best compromise between piece‐wise constant regions of averaged voxel susceptibility and sharp features appearing at edges of tissues with different susceptibility. While the closed form solution appeared always over‐smoothed compared to TV‐MEDI, the ℓ_2_‐MEDI showed a high variance of susceptibility values inside trabecular bone regions, independent of the regularization parameter (see Supporting Information Figure S2. Besides this visual comparision, the regression of the quantitative values in the drawn ROIs in the subtalar and the tuber calcanei of all volunteers showed the highest correlations with the TV‐MEDI regularization (compare Figure 4 and Supporting Information Figure S2). In the regularization of the performed MEDI, an important part of the regularizer is a morphological edge mask derived from magnitude information that ensures that susceptibility is piece‐wise constant and not artificially smoothed over true discontinuities. Due to magnitude modulations in trabecular bone regions it is possible that the used edge detection algorithm identifies edges in these regions where susceptibility varies continuously without large discontinuities. The down‐weighted regularization in falsely detected edge voxels inside trabecular bone can therefore alter the measured susceptibility. We found that within an order of magnitude of the regularization parameter the chosen number of edge voxels inside the whole imaging volume did not change the resulting susceptibility reconstructions significantly (not shown). The comparison of MEDI to the implemented closed‐form solution for dipole inversion, where no edge information is incorporated, shows that the susceptibility contrast is not diminished by the heuristic choice of an edge detection threshold. The same comparison also shows that the chosen data weighting term *W*_d_ in (1) is of less importance as the absence of any data weighting in the closed‐form solution also leads to comparable susceptibility contrast. As the susceptibility gradient in trabecularized bone marrow is expected to be smoothly varying, a total generalized variation regularizer could mitigate possible stair‐case artefacts of linear susceptibility gradients. However, in this work we limited the class of regularizers under investigation to the more common total variation.

For the nonlinear TV‐MEDI we used Nesterov's algorithm (NESTA),^53^ which results in fast and accurate reconstruction without the need to define any split variables as in Split‐Bregman algorithm. Despite the high resolution and the relatively large FOV the algorithm converged in well under three minutes with a MATLAB implementation on a currently standard lap top machine (4 GHz processor, 16 GB RAM), while similar implementation of Gauss‐Newton algorithms can take up to an hour on the same machine.

The numerical simulation in the trabecular bone model of randomly distributed overlapping spherical susceptibility inclusions inside a cubic box confirm the feasibility of QSM to measure changes in the trabecular bone density. Independent of the assumed susceptibility difference between bone inclusions and the outside medium, the susceptibility average over the box could be recovered from the forward simulated field map by only a simple direct solution of to field‐to‐susceptibility inversion not incorporating any morphological edges or signal weights. Even though inside the ROI the averaged frequency shift from the bone inclusions is small,^6^ the bulk effect of the ROI to the surrounding is sufficient for the global dipole inversion step to be sensitive on the distribution of susceptibility sources. In bone literature focusing on $R_{2}^{*}/R_{2}^{\prime }$‐mapping in trabecular networks, several other trabecular bone models have been discussed. While an extended treatment of such models is beyond the present work, the more simple approach used here to model a voxel with trabecularized bone marrow was however successful in confirming the sensitivity of QSM on BV/TV.

A concise comparision of the slopes from the regression of susceptibility versus apparent BV/TV in vivo and the corresponding simulation results is not possible at this point. The direct comparison of the BV/TV‐to‐*χ* slope from the in vivo scans (Figure 4) shows that in the calcaneus the measured *χ* TV‐MEDI varies about twice as fast with apparent BV/TV than the steepest simulated BV/TV‐to‐*χ* slope (Δ*χ* = −2 ppm) in Figure 8. While this can suggest that the true susceptibility of trabecular bone might be greater than the values simulated, other properties differ between the simulation and the in vivo measurements that render such interpretations uncertain. In the literature, the range of reported susceptibility values for bone is fairly broad; While several studies from the 1990's based on $R_{2}^{\prime }$‐imaging report values around −0.3 ppm for trabecular bone referenced to water at 0 ppm,^58^, ^59^ more recent studies also based on QSM have reported much more diamagnetic values around −2 ppm and lower with respect to water.^5^, ^21^, ^60^, ^61^, ^62^ The bone marrow in the calcaneus consist of 100% fat with a reported susceptibility difference to water of ∼0.6 ppm.^59^, ^63^ Assuming a true trabecular susceptibility at the higher end of the reported values and taking the fatty susceptibility source of the inter‐trabecular fatty bone marrow into account, one could argue for a steeper in vivo BV/TV‐to‐*χ* slope than in the presented numerical simulation. However, the different dipole inversion methods, the presence of noise, the simplified trabecular network topology in the simulated model and the known over‐estimation of the apparent BV/TV in the gradient‐echo based measurement together with a low number of in vivo samples are all factors that do not allow a detailed interpretation of the presented results with respect to the true value of trabecular bone susceptibility.

The observed sensitivity of QSM to trabecular bone density in the calcaneus, is however well explained by the strongly simplified trabecular bone model. A detailed comparison of QSM to $R_{2}^{*}$‐mapping is out of the scope of this work, but from Figure 8 one can also appreciate two theoretical advantages of QSM over an $R_{2}^{*}$. $R_{2}^{*}$ is inherently dependent on the field strength and also on the voxel size, observable in the upper plot in Figure 8 as the nonlinear increase of $R_{2}^{\prime }/f_{0}$ with BV/TV, whereas QSM algorithms incorporate both properties as input and measured bulk susceptibility can therefore be independent on the field strength and voxel size. While a theoretical investigation complemented with phantom experiments are necessary in future work to further address possible (dis)advantages of QSM over $R_{2}^{*}$, the study presented here is able to show sensitivity of QSM on trabecular bone density. Further work is necessary in order to investigate whether bone marrow QSM can probe in vivo trabecular density changes induced by pathological bone loss or osteoporosis.

While all implemented regularizations were able to significantly detect BV/TV differences, the current preliminary study has several limitations.

A fundamental issue with QSM is its inability to produce absolute susceptibility values. Starting from MR phase information which is inherently difficult to reference to an absolute value—particularly in parallel imaging using multiple coils—the QSM processing also shows this limitation. In the solution of the ill‐posed dipole inversion deconvolution, the convolution kernel's center singularity is re‐normalized by the Lorentz sphere contribution, which results in a zero DC offset of the dipole kernel in k‐space.^64^ Consequently, the dynamic range of resulting susceptibility maps is centered around the average k‐space energy of the local field map before dipole inversion, which is highly dependent on object properties and therefore varying between subjects. Brain QSM studies therefore introduced a referencing procedure, where a specific ROI thought to be relatively homogeneous and of comparable size between subjects is defined and the susceptibility map are centered around the mean susceptibility value inside the ROI. There is an ongoing debate^44^ about which reference tissue is best suited for comparing QSM values across different brain subjects in a study.^65^ While cerebrospinal fluid or white matter in the internal capsule of the brain can serve well as a reference tissue for QSM, in the body only subcutaneous fat was proposed. In the abdominal region subcutaneous fat is more homogeneous and can be used to reference susceptibility maps between subjects. However as can be seen in bSSFP scans (Figure 4) the subcutaneous tissue is highly inhomogenous and susceptibility is unlikely to be constant over several voxels. Hence, QSM referencing is difficult in the ankle region.

Another limitation of the current study is the dependence of susceptibility reconstructions on the regularization parameter λ in (1). This parameter was optimized by visual comparison of reconstructions on a wide range of λ values and validated by an L‐curve heuristic in only one subjects, shown in Supporting Information Figure S2. The obtained regularization parameter was then used for the QSM dipole inversion in all other datasets. While this procedure followed multi‐subject studies in brain QSM, where the size of the cropped out brain in relation to the FOV is not largely varying, for the imaging of the ankle, the ratio of the imaging object to the FOV can vary significantly as with constant FOV, subjects can have different foot sizes. By inspection of Equation (1) one can see that the regularization parameter depends not only on the voxel size (through the gradient operator in the regularizer), and the edge weighting *W*_g_, but also on precisely the ratio between tissue voxel to background/air voxels in the FOV. Therefore, the optimal regularization parameter in one subject may differ for other subjects with different foot sizes.

A more crucial limitation is the accuracy of the apparent BV/TV measured with the bSSFP sequence. This measurement resembles the high‐resolution imaging approach to access trabecular bone density. The formed gradient echoes to image trabeculae are susceptible to chemical shift dispersion and off‐resonance effects near the trabeculae–bone marrow interface. Both effects lead to blurring and artificially enlarged trabeculae. The actual BV/TV is therefore systematically smaller than the apparent BV/TV from the bSSFP measurement.^5^ Trabecular bone imaging based on spin‐echo sequences does not suffer from the artifactually increased BV/TV, but needs considerably larger repetition times resulting in longer scan time and less motion robustness. From the complementary highly significant correlations of QSM with the direct CT attenuation as well as the indirect measure $R_{2}^{*}$ of trabecularization—both known to have high correlation with actual BV/TV^7^, ^56^one can deduce that QSM is also sensitive to the trabecular bone density.

In support of reproducible research, source code for the QSM post‐processing, ROI analysis, and figure reproduction scripts are available for download at https://github.com/maxdiefenbach/trabecular_bone_QSM.git (SHA‐1=f0df3fbde928da49c04d91e40c1a6b7c60245696). The git repository also includes an exemplary reconstructed MRI source datasets used in this work.

## 5 CONCLUSION

In conclusion, the present work developed a methodological framework to simultaneously measure $R_{2}^{*}$ and susceptibility in trabecularized yellow bone marrow, addressing the presence of fat. The presented preliminary results showed a correlation between measured susceptibility values and CT attenuation, apparent BV/TV and $R_{2}^{*}$ 7 demonstrating the sensitivity of QSM on trabecular bone density.

## ACKNOWLEDGMENT

The present work was supported by the European Research Council (grant agreement No 677661—ProFatMRI) and Philips Healthcare. We also acknowledge the use of the MEDI toolbox^52^ made freely available at http://www.weill.cornell.edu/mri/pages/qsm.html and a NESTA implementation from.^53^ The authors gratefully acknowledge the Gauss Centre for Supercomputing e.V. (www.gauss‐centre.eu) for funding this project by providing computing time on the GCS Supercomputer SuperMUC at Leibniz Supercomputing Centre (www.lrz.de).

## Conflict of Interest

Dr. Jakob Meineke is an employee of Philips Healthcare.

## Supporting information


**Figure S1** Water–fat signal model **Figure S2** Top: heuristic L‐curve, discreprancy $\left|\right|\left(F^{†}DF\chi_{\text{est}}\left(\lambda \right)-f_{L}\right){\left|\right|}_{2}^{2}$ versus regularization parameter λ (compare to Equation (1)) for all three implemented dipole‐inversion schemes. Bottom: susceptibility maps corresponding to different λ's **Figure S3** Regression analysis of ROI label statics, TIMGRE parameters $R_{2}^{*}$ and susceptibility *χ* versus the apparent BV/TV estimated from the bSSFP scan for the two additional dipole‐inversion schemes, the ℓ_2_‐regularized closed form solution and ℓ_2_‐MEDI (one outlier removed). Compare to Figure 4 from the main text **Figure S4** Extended version of Figure 5 from the main text showing slices of all main anatomical planes for each patient dataset **Figure S5** Regression analysis of ROI label statics, TIMGRE parameters $R_{2}^{*}$ and susceptibility *χ* versus the CT attenuation for the two additional dipole‐inversion schemes, the ℓ_2_‐regularized closed form solution and ℓ_2_‐MEDI. Compare to Figure 6 from the main text.
