# Supplementary material for: On the sensitivity of quantitative susceptibility mapping for measuring trabecular bone density
Source: Magn Reson Med. 2018 Sep 28;81(3):1739–54. doi: 10.1002/mrm.27531 (PMC6585956; doi:10.1002/mrm.27531)
Supplement: Supplementary file 1 — Figure S1 Water–fat signal model Figure S2 Top: heuristic L‐curve, discreprancy ||(F†DFχest(λ)−fL)||22 versus regularization parameter λ (compare to Equation (1)) for all three implemented dipole‐inversion schemes. Bottom: susceptibility maps corresponding to different λ's Figure S3 Regression analysis of ROI label statics, TIMGRE parameters R2* and susceptibility χ versus the apparent BV/TV estimated from the bSSFP scan for the two additional dipole‐inversion schemes, the ℓ2‐regularized closed form solution and ℓ2‐MEDI (one outlier removed). Compare to Figure 4 from the main text Figure S4 Extended version of Figure 5 from the main text showing slices of all main anatomical planes for each patient dataset Figure S5 Regression analysis of ROI label statics, TIMGRE parameters R2* and susceptibility χ versus the CT attenuation for the two additional dipole‐inversion schemes, the ℓ2‐regularized closed form solution and ℓ2‐MEDI. Compare to Figure 6 from the main text. [file MRM-81-1739-s001.pdf]

Supporting Information: "On the Sensitivity of Quantitative Susceptibility Mapping for Measuring Trabecular Bone Density"

Contents

- Supporting Information S1: Water–fat signal model
- Supporting Information Figures
  - Supporting Information Figure S2: Top: heuristic L-curve, discreprancy $||(F^{\dagger}DF\chi_{\text{est}}(\lambda) - f_L)||_2^2$ versus regularization parameter λ (compare to Equation [1]) for all three implemented dipole-inverion schemes. Bottom: susceptibility maps corresponding to different λ's.
  - Supporting Information Figure S3: Regression analysis of ROI label statics, TIMGRE parameters R_2^* and susceptibility χ versus the apparent BV/TV estimated from the bSSFP scan for the two additional dipole-inverion schemes, the ℓ_2-regularized closed form solution and ℓ_2-MEDI (one outlier removed). Compare to Figure 4 from the main text.
  - Supporting Information Figure S4: Extended version of Figure 5 from the main text showing slices of all main anatomical planes for each patient dataset.
  - Supporting Information Figure S5: Regression analysis of ROI label statics, TIMGRE parameters R_2^* and susceptibility χ versus the CT attenuation for the two additional dipole-inverion schemes, the ℓ_2-regularized closed form solution and ℓ_2-MEDI. Compare to Figure 6 from the main text.

Supporting Information S1: Water–fat signal model

The evolution of the complex MR signal in a voxel sampled at the n-th echo time, $\hat{s}_n \equiv \hat{s}(t_n), n = 1, ..., N$, is given by

$$\begin{aligned} \hat{s}_n &= (W + c_n F)\, e^{(i2\pi f_B - R_2^*)t_n}, \\ c_n &= \sum_{p=1}^{P} a_p e^{i2\pi \Delta f_p t_n}, \quad \text{with} \quad \sum_{p=1}^{P} a_p = 1, \end{aligned} \quad [1]$$

where $W, F \in \mathbb{C}$ are the complex signals of water and fat, respectively, that both share a common transverse relaxation rate R_2^*. f_B is the averaged magnetic field in the voxel. The fat spectrum is fixed by P spectral peaks of corresponding relative amplitudes a_p and chemical shifts Δf_p (1). For the field mapping step in Quantitative Susceptibility Mapping in the presence of fat, for each voxel one solves the following optimization problem:

$$\beta = \arg\min_{\beta'} \left(\sum_n |s_n - \hat{s}_n(\beta')|^2 \right)^{1/2}, \quad [2]$$

where s_n is the measured voxel signal and echo time t_n and the parameters vector is defined by $\beta = [W, F, f_B, R_2^*]^T$. The proton density weighted fat fraction (PDFF) is obtained by

$$\text{PDFF} = \begin{cases} 1 - |W|/|W + F| & \text{for } |F| \leq |W| \\ |F|/|W + F| & \text{for } |F| > |W|, \end{cases} \quad [3]$$

which reduces noise bias (2). Consequently, the proposed method yields multi-parametric three-dimensional parameter maps: PDFF, f_B, and R_2^*. The field map f_B was subject to further QSM processing. There were no additional corrections made to the R_2^* map.

Supporting Information Figures

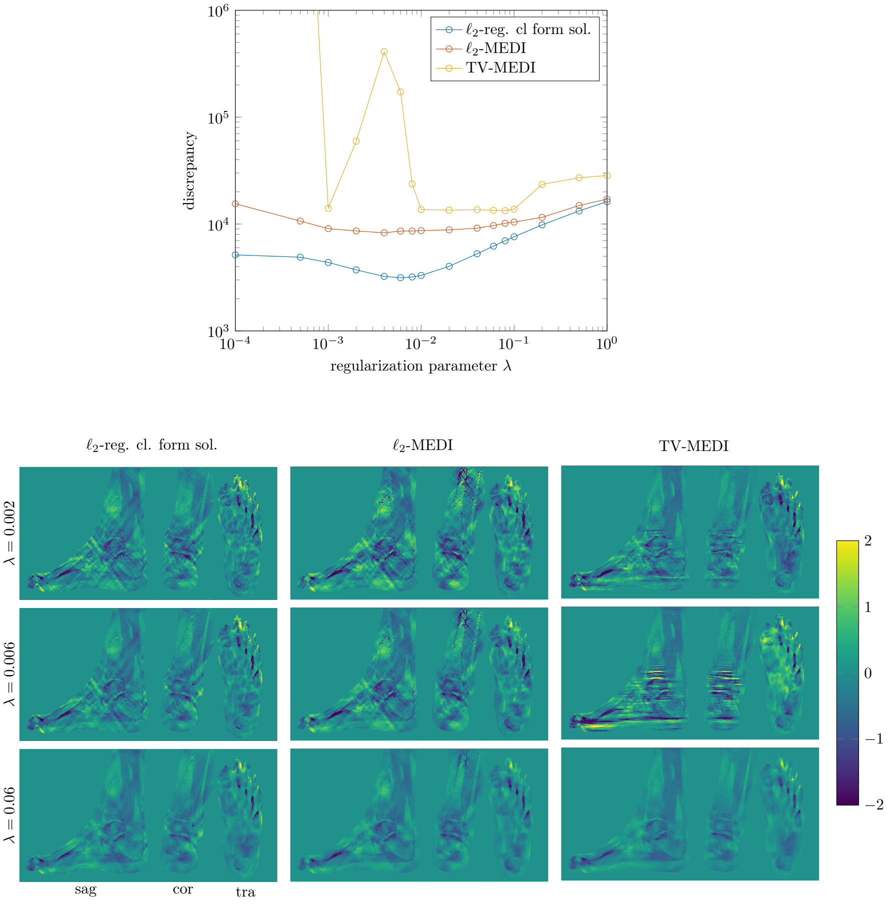

Supporting Information Figure S2: Top: heuristic L-curve, discreprancy $||(F^\dagger D F \chi_{\text{est}}(\lambda) - f_L)||_2^2$ versus regularization parameter λ (compare to Equation [1]) for all three implemented dipole-inverion schemes. Bottom: susceptibility maps corresponding to different λ's.

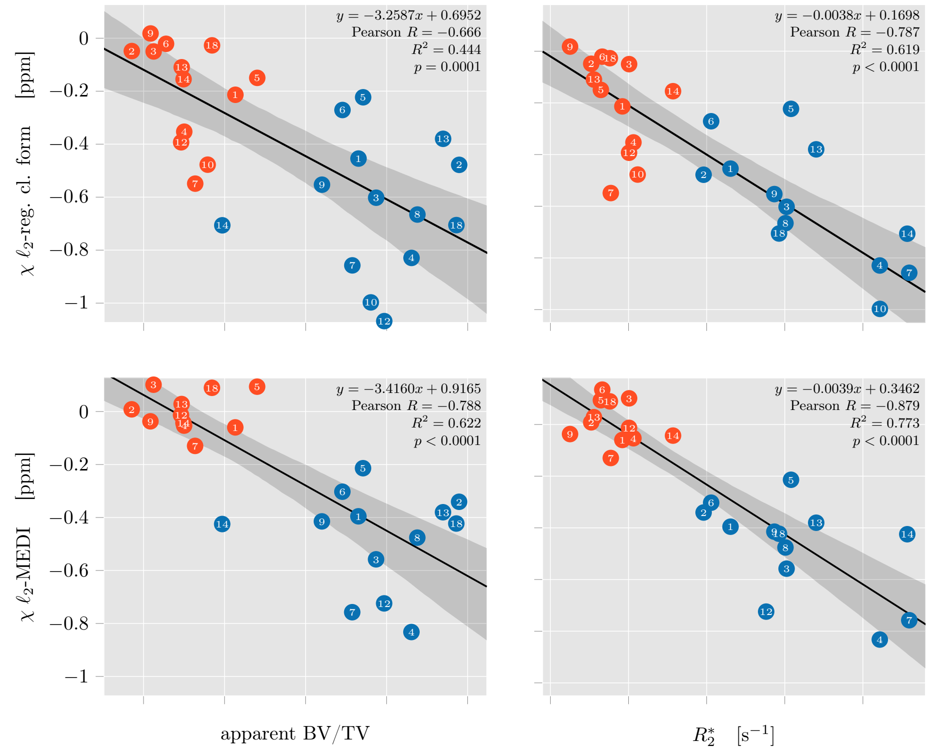

Supporting Information Figure S3: Regression analysis of ROI label statics, TIMGRE parameters R_2^* and susceptibility χ versus the apparent BV/TV estimated from the bSSFP scan for the two additional dipole-inverion schemes, the ℓ_2-regularized closed form solution and ℓ_2-MEDI (one outlier removed). Compare to Figure 4 from the main text.

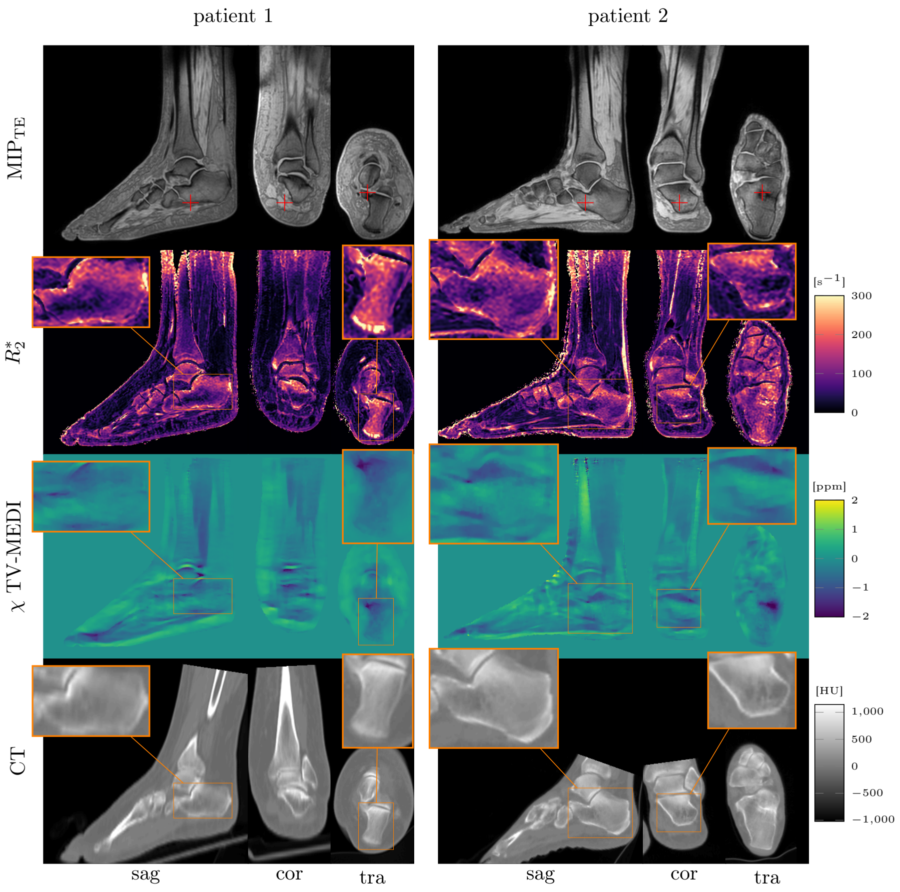

Supporting Information Figure S4: Extended version of Figure 5 from the main text showing slices of all main anatomical planes for each patient dataset.

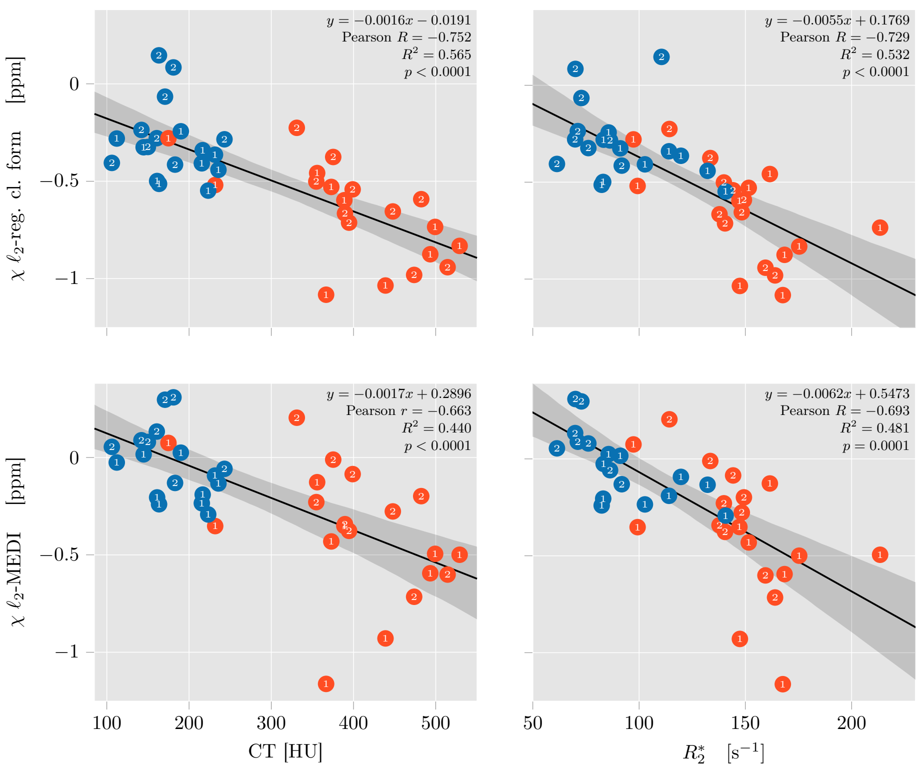

Supporting Information Figure S5: Regression analysis of ROI label statics, TIMGRE parameters R_2^* and susceptibility χ versus the CT attenuation for the two additional dipole-inverion schemes, the ℓ_2-regularized closed form solution and ℓ_2-MEDI. Compare to Figure 6 from the main text.

References

1. Yu H, Shimakawa A, McKenzie CA, Brodsky E, Brittain JH, Reeder SB. Multiecho water-fat separation and simultaneous r2* estimation with multifrequency fat spectrum modeling. Magnetic Resonance in Medicine 2008; 60:1122–1134.

2. Liu CY, McKenzie CA, Yu H, Brittain JH, Reeder SB. Fat quantification with ideal gradient echo imaging: Correction of bias fromt1 and noise. Magnetic Resonance in Medicine 2007; 58:354–364.
